# Ultra-precise detection of mutations by droplet-based amplification of circularized DNA

**DOI:** 10.1186/s12864-016-2480-1

**Published:** 2016-03-09

**Authors:** Kaile Wang, Qin Ma, Lan Jiang, Shujuan Lai, Xuemei Lu, Yali Hou, Chung-I Wu, Jue Ruan

**Affiliations:** Key Laboratory of Genomic and Precision Medicine, Beijing Institute of Genomics, Chinese Academy of Sciences, Beijing, China; University of Chinese Academy of Sciences, Beijing, China; State Key Laboratory of Biocontrol, School of Life Sciences, Sun Yat-Sen University, Guangzhou, China; Department of Ecology and Evolution, University of Chicago, Illinois, USA; Agricultural Genomics Institute at Shenzhen, Chinese Academy of Agricultural Sciences, Shenzhen, China

**Keywords:** Accurate sequencing, Low frequency mutation detection, Low bias amplification, Droplet (water in oil) based amplification, Low input NGS library

## Abstract

**Background:**

NGS (next generation sequencing) has been widely used in studies of biological processes, ranging from microbial evolution to cancer genomics. However, the error rate of NGS (0.1 % ~ 1 %) is still remaining a great challenge for comprehensively investigating the low frequency variations, and the current solution methods have suffered severe amplification bias or low efficiency.

**Results:**

We creatively developed Droplet-CirSeq for relatively efficient, low-bias and ultra-sensitive identification of variations by combining millions of picoliter uniform-sized droplets with Cir-seq. Droplet-CirSeq is entitled with an incredibly low error rate of 3 ~ 5 X 10^-6^. To systematically evaluate the performances of amplification uniformity and capability of mutation identification for Droplet-CirSeq, we took the mixtures of two *E. coli* strains as specific instances to simulate the circumstances of mutations with different frequencies. Compared with Cir-seq, the coefficient of variance of read depth for Droplet-CirSeq was 10 times less (*p* = 2.6 X 10^-3^), and the identified allele frequency presented more concentrated to the authentic frequency of mixtures (*p* = 4.8 X 10^-3^), illustrating a significant improvement of amplification bias and accuracy in allele frequency determination. Additionally, Droplet-CirSeq detected 2.5 times genuine SNPs (*p* < 0.001), achieved a 2.8 times lower false positive rate (*p* < 0.05) and a 1.5 times lower false negative rate (*p* < 0.001), in the case of a 3 pg DNA input. Intriguingly, the false positive sites predominantly represented in two types of base substitutions (G- > A, C- > T). Our findings indicated that 30 pg DNA input accommodated in 5 ~ 10 million droplets resulted in maximal detection of authentic mutations compared to 3 pg (*p* = 1.2 X 10^-8^) and 300 pg input (*p* = 2.2 X 10^-3^).

**Conclusions:**

We developed a method namely Droplet-CirSeq to significantly improve the amplification bias, which presents obvious superiority over the currently prevalent methods in exploitation of ultra-low frequency mutations. Droplet-CirSeq would be promisingly used in the identification of low frequency mutations initiated from extremely low input DNA, such as DNA of uncultured microorganisms, captured DNA of target region, circulation DNA of plasma et al, and its creative conception of rolling circle amplification in droplets would also be used in other low input DNA amplification fields.

**Electronic supplementary material:**

The online version of this article (doi:10.1186/s12864-016-2480-1) contains supplementary material, which is available to authorized users.

## Background

Massive parallel DNA-sequencing technologies have dramatically improved the researches in genetics, biomedicine, cancer evolution, and the other significant and intriguing fields in life science. However, an inevitable error rate of NGS (next generation sequencing) approaches ascribing to library preparation, sequencing et al. [1], remains certainly high, ranging from 0.1 % to 1 % varied in disparate platforms [2–4] and data processing strategies [4, 5]. Although the error rate presents feasibly tolerable in the case of studying high frequency mutations [6, 7], it severely obscures the precise determination of low frequency mutations [8]. Taken a specific example, in the promising field of cancerous evolution, the sequencing errors arisen from NGS platform could be perfectly controlled in the case of studying germline mutations or high frequency somatic mutations [6, 7], however, they particularly hinder the unambiguous inference of the evolutionary trajectories of the cancer subclones [9] due to the obscures of low frequency mutations.

Additionally, precise calibration of genetic heterogeneity is well acknowledged as the prerequisite to understand the development and evolution of species and cancers. The de novo somatic mutations of offspring substantially facilitate the revelation of the genetic dynamics underlying biological processes [10–12], accurately evaluate the rates of spontaneous mutations [13, 14], and exquisitely profile the developmental trajectories of cell linages [15–17]. Furthermore, cascades of pioneering studies have confirmed that somatic mutations play critical roles in the formation of tumor heterogeneity [17–19] and drug-resistance [20, 21]. Nevertheless, majority of somatic mutations, unfortunately, has been characterized by low or ultra-low frequency [8], which obscures their precise identification because of lacking of appropriate methods. Theoretically, “abundantly deep sequencing” could address this problem and detect all, or at least most, of the mutations with low or ultra-low frequency, however, it can’t completely avoiding inherent PCR and sequencing errors and is barely feasible from the economical point of view [22, 23]. As long as for a reasonable depth after compromising on costs and accuracy, it would always suffer from the bewilderment to distinguish very low frequency mutations and sequencing errors (such as those below 1 %) [1, 10, 24].

Thereby, detecting the low or ultra-low frequency mutations based on NGS platform appears intriguing but still remains incredibly challenging. Great efforts have been substantially made to develop exquisite methods addressing this problem [22–35]. Majority of the methods utilizes unique barcodes (or tags) to eliminate PCR and sequencing errors [8, 30–33], like Safe-seq, Duplex-seq. These methods tag every target molecule with different barcodes (random bases) and send them for sequencing. The produced reads with identical barcodes are regarded as one “read family” and are used to eliminate the PCR and sequencing errors. However, the efficiency of these methods relies heavily on the read number of each “read family”. To obtain high precision, one molecule should be sequenced many times; this constrains the application of these methods, especially for large genome analyses. Cir-seq (circle sequencing) tandems the replicates of one single-strand circularized molecule by RCA (rolling circle amplification) [34, 35] to detect a tag-free read family. The original molecule can be sequenced at least twice by a pair of PE (pair end) reads by controlling the original DNA fragment sizes; A pair of PE reads is termed as one “read family”. This method effectively overcomes the disadvantage of the barcode methods. However, RCA inevitably introduces amplification bias, thus, greatly limiting the application of this method, especially when the input DNA is low, such as for DNA circulating in plasma or serum, forensic DNA, DNA of uncultured microorganisms, DNA of tiny cancer sub-clones, and ancient, degenerated DNA. The difficulty of RCA bias is the same as that for whole genome amplification using MDA (multiple displacement amplification) when the input DNA is low.

In this study, we introduced an innovated method termed Droplet-CirSeq, which combines millions of pico-liter droplets and the Cir-seq for uniform and ultrasensitive detection of rare mutations. Compared with Cir-seq, Droplet-CirSeq greatly reduced the amplification bias, represented more authentic allele frequency and detected rare mutations more effectively and precisely. Droplet-CirSeq was entitled with an incredibly low error rate of 3 ~ 5 X 10^-6^. According to the systematical assessment, Droplet-CirSeq detected 2.5 times genuine SNPs more than Cir-seq and had a 2.8-fold lower false positive rate and a 1.5-fold lower false negative rate when the input was 3 pg of circularized DNA.

## Methods

### Droplet-CirSeq library preparation

Genomic DNA (1 ~ 3 μg) was sheared into 100 ~ 200 bp fragments in Buffer AE (10 mM Tris-Cl, 0.5 mM EDTA) using Covaris S220 in 100 μl volume (shearing condition: duty cycle: 10 %, intensity: 5, cycles per burst: 100, time: 600 s), then purified with the 1.8X Ampure XP beads. The purified DNA was phosphorylated at 37 °C for 30 min in a standard reaction consisting of 44 μl DNA, 10U T4 PNK (T4 Polynucleotide Kinase, NEB, M0201S), 50 mM Tris-HCl pH 7.5, 10 mM MgCl_2_, 1 mM ATP, 10 mM DTT, then was run on a 4 % agarose gel at 80 V for 80 min. Subsequently, the gels with DNAs in length of 80 ~ 140 bp marked with 20 bp DNA ladder (Takara) were particularly cut off, and further extracted using QIAGEN MinElute Gel Extraction Kit (6X buffer QG). The ultimate DNA concentration was calibrated using Qubit 2.0 dsDNA HS Assay kit.

A column of 20 μl DNA (about 100 ng) was denatured at 95 °C for 3 min, and immediately put on ice for another 3 min, then added by a mixture of 2.5 μl 10X Cirlagase buffer, 1.25 μl 50 mM MnCl_2_, 1.25 μl Cirligase (Epicentre CL9025K). The mixtures were further incubated at 60 °C for 2 h, and the inner enzymes were inactivated by heating at 80 °C for 10 min. Subsequently, 0.5 μl Exonuclease I (NEB, M0293S) and 0.5 μl Exonuclease III (NEB, M0206S) were added into the reaction and jointly incubated at 37 °C for 1 h. The inner enzymes were inactivated at 80 °C for another 20 min. The successfully circularized DNA was purified using the QIAquick Nucleotide Removal Kit (10X PN1) and its final concentration was calibrated using Qubit® ssDNA Assay Kit.

A column of 2.5 μl circularized DNA was placed into 0.2-ml tubes containing 5 μl 10X phi29 DNA Polymerase Reaction Buffer (NEB, M0269S), 2.5 μl exonuclease-resistant hexamer (500 μM), 2.5 μl 10 mM dNTP, 1 μl 100XBSA, 27 μl ddH_2_O, which was denatured at 95 °C for 3 min, then put on ice immediately for another 3 min, and further added by a mixture of 1 μl UDG (uracil-DNA glycosylase, NEB, M0280S), 1 μl Fpg (formamidopyrimidine DNA glycosylase, NEB, M0240S) and 2.5 μl phi29 DNA Polymerase (NEB, M0269S). The thoroughly mixed 45 μl RCA reaction and 5 μl stabilizer were put into *RainDrop Source chip (RainDance Technologies*, *RainDrop Digital PCR System)* together to produce water-in-oil emulsion droplets as the manufacturer’s recommendations. In general, there will be 5 ~ 10 million droplets per 50 μl volume formed in about an hour. The droplets containing RCA mixture were completely reacted at 30 °C for 8 ~ 16 h, and the inner enzymes were further inactivated at 65 °C for 10 min. The emulsion was broken using PFO (1H, 1H, 2H, 2H, - Perflurooctanol) to recover the amplified DNA as follows: adding 2 ~ 3 volume of PFO (100 ~ 150 μl) to the reaction mixture and admixing thoroughly, incubating at room temperature for 5 min, then centrifuging at 3000 rpm for 5 min. The supernatant was then purified with 1.8X Ampure XP beads. The recovered DNA can be used for preparing standard NGS libraries.

### Standard NGS library preparation

The amplified DNA (about 1 μg) was sheared into 700 bp in ddH_2_O using Covaris S220 at 85 μl volume (shearing condition: duty cycle: 5 %, intensity: 3, cycles per burst: 200, time: 75 s), then end-repaired using NEBNext® End Repair Module (NEB, E6050S) and purified with 1X Ampure XP beads. The NEBNext® dA-Tailing Module (NEB, E6053S) was used to dA-tailing. 1X Ampure XP beads were used to clear up the reaction mixture. Ligation was performed at 20 °C for 30 min using NEBNext® Quick Ligation Module (NEB, E6056S), 1 μl barcode adaptor (Bioo Scientific, NEXTflex™ DNA Barcodes, 514102) was used. The production was purified with 1X Ampure XP beads and run on a 2 % agarose gel to perform the size-selection. The gel with DNA in length of 500 ~ 700 bp was cut off and further extracted using QIAGEN MinElute Gel Extraction Kit (3X buffer QG). The ultimate DNA concentration was calibrated using Qubit 2.0 dsDNA HS Assay kit. PCR was performed in a reaction consisting of 24 μl ligated DNA (30 ng) and ddH_2_O, 1 μl NEXTflex™ Primer Mix (Bioo Scientific, 514102), 25 μl 2x Phusion Master Mix with HF Buffer (Thermo Scientific, F531) as the following cycling conditions: 98 °C for 2 min and 10 cycles of 98 °C for 30s, 65 °C for 30 s, and 72 °C for 1 min, then 72 °C for 4 min and held at 4 °C.

### Droplet-CirSeq data processing

The data processing of Droplet-CirSeq and Cir-seq reads is quite different from that of regular re-sequencing data. First, a consensus sequence should be determined from multiple tandem copies of circular DNA within a pair of PE read. Second, the break point of circularized DNA should be accurately detected by mapping it to the reference genome.

The consensus sequences (CSs) can be determined by aligning Read1 and Read2 from the same pair of PE reads with a mismatch rate of approximately 0.05. The detailed procedure of CSs determination was summarized as following. Firstly, the Read1 and the reverse complement of Read2 are literally merged into one longer read according to their status of overlapping. The merged read contains copies of circularized DNA. Secondly, the merged reads are aligned against itself to determine the smallest unit of the circle. Simultaneously, the base quality of the consensus sequence is scored according to its exact number and sequencing quality. For instance, if a site in the CSs is occasionally sequenced only once, a severe penalty would be assigned and thus discarded in the following process of variant calling. In case of a site with different bases in distinct copies, it is also considered to have a severe penalty and discarded.

The CSs were mapped onto the reference genome using Bwa [36]. Since every position of the CSs could be the correct junction of the linear DNA sequence, we iteratively assign every base of the CSs as the start point of the linear DNA sequence and independently mapped them onto the reference genome, where the one with the least number of mismatches and INDELs (small insertions and deletions) was chosen as the original linear DNA sequence, and further was utilized to call variants.

The source code of Droplet-CirSeq data processing is available on: http://sourceforge.net/projects/Droplet-Seqcode/files/Droplet-code/.

### Standard NGS data processing

The 2X150 PE reads were mapped to the reference genome using Bwa, The variants in terms of SNPs and INDELs were called using bcftools view with options of –bvcg. The variants were further filtered by quality (QUAL = 222) and a ratio of reference bases over alternative bases (<0.01).

## Results

### Library construction and sequencing for Droplet-CirSeq

**Fig. 1:**
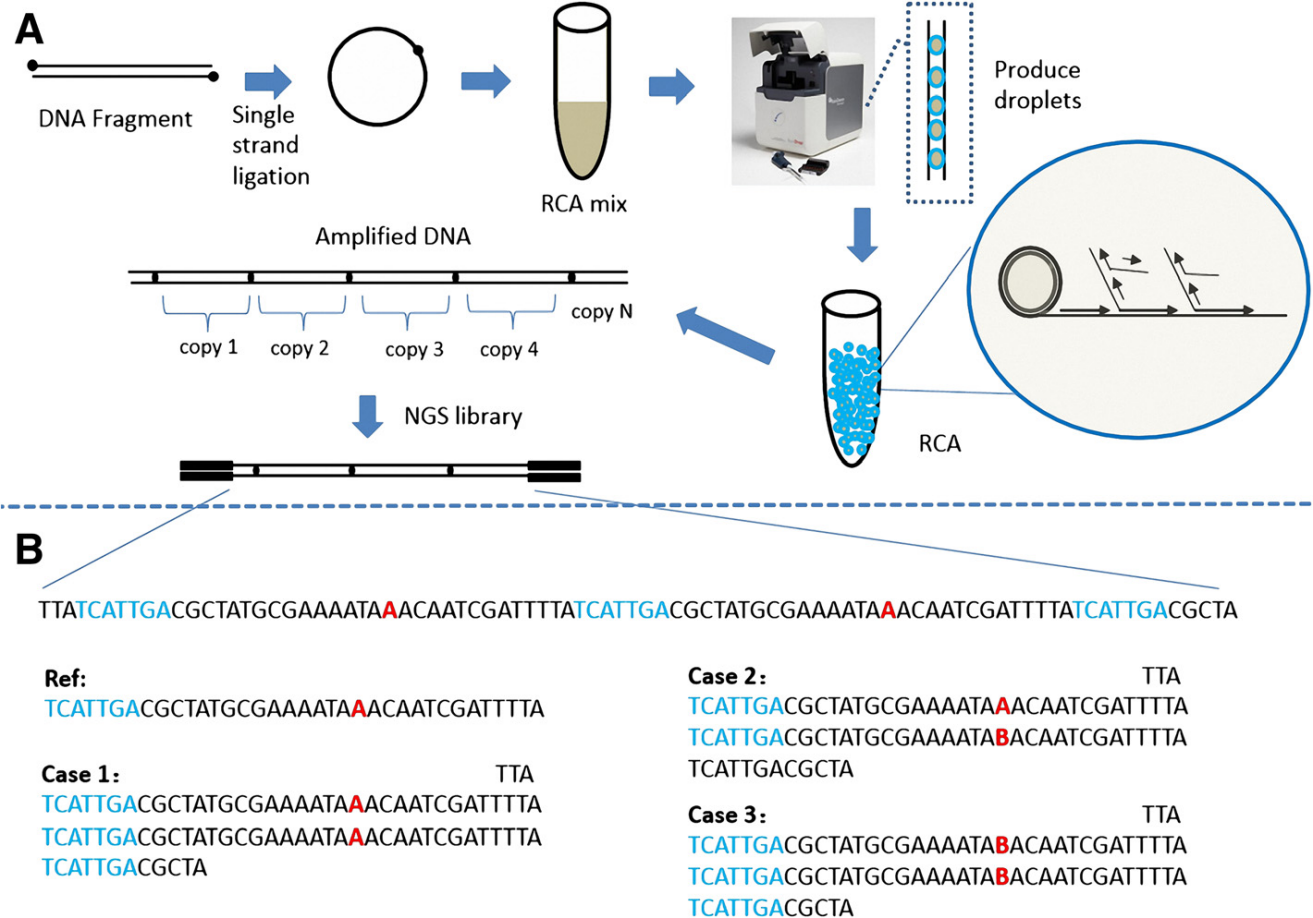
Overview of Droplet-CirSeq. **a** Droplet-CirSeq workflow. Genomic DNA was sheared into fragments shorter than half the length of the sequencing read and then denatured into single-stranded DNA molecules that were circularized using single-strand DNA ligase. After eliminating the linear DNA using DNA exonucleases, the circularized single-stranded DNA was used for RCA (rolling circle replication). The circularized DNA and RCA reaction mix was added to a RainDrop Source chip to produce water-in-oil emulsion droplets. Generally, approximately 5 ~ 10 million droplets formed in approximately an hour in a 50 μl volume. The droplets containing RCA mix were allowed to continue to react for 4 ~ 16 h in order to amplify enough DNA for standard NGS library preparation in the following steps. Please note that the insert size of the standard NGS libraries must be larger than twice the length of the original circularized DNA to avoid sequencing the same DNA copy twice instead of sequencing two independent-amplified copies. **b** Error correction. Here is an example to explain the error correction strategy. Multiple copies of the original circularized DNA were examined in every PE read. “A” (red color) represents the base, which may have errors generated during PCR or sequencing. There will be three cases present in the sequencing result: AA (case 1), no errors; AB (case 2), one read error; and BB (case 3), two read errors. B stands for T/C /G. In the following bioinformatics analysis, Case 2 and Case 3 will be filtered except when BB has the same bases, such as TT, GG, or CC (false positive)

The strategies of library construction and sequencing for our Droplet-CirSeq were schematically illustrated in Fig. 1. As it elucidates, the genomic DNA was sheared into small fragments, which were shorter than the length of a single PE read, to ensure that each fragment could be sequenced at least twice in a pair of PE reads. Such a strategy that each fragment is repeatedly sequenced eliminates PCR error and sequencing error through exquisite data process (Fig. 1). These fragments were denatured into single-strand molecules and then circularized by single-strand DNA ligase. The circularized single-strand DNA fragments were amplified independently in 5 ~ 10 million water-in-oil emulsion droplets using phi 29 DNA polymerase with random primers. In our study, we utilized the RainDrop Source of *RainDrop Digital PCR System* to produce the millions of picoliter droplets because of its competitive characteristic of generating droplets with extremely uniform dimensions. After amplification, the emulsion droplets were broken using 1H, 1H, 2H, 2H-perfluorooctane to collect the amplified DNA, which was composed of tandem copies of the sheared DNA fragments. The purified DNA can be easily embraced into the standard protocols of NGS library construction on a broad variety of platforms, including the Illumina Hiseq and Miseq, and Life technologies Ion PGM, et al. Of note, it is importantly emphasized that the insert sizes of the standard NGS library is required to be at least twice longer than that of the original circularized DNA, which guarantees the circularized DNA was sequenced from different tandem copy units.

In our case, based on the purified DNA, we constructed the Droplet-CirSeq library by stringently following the protocol of Droplet-CirSeq library preparation (Methods), and generated 2X150 bp PE reads using the Hiseq 2500 platform. The PE reads were first used to detect the CSs after filtering out the low quality reads, and the CSs were then rotation-insensitively mapped to the reference genome to determine the junction sites of the circularized DNA. The correctly mapped CSs were used to accurately identify the mutations. As comparison, another method, Cir-seq was simultaneously implemented according to the abovementioned procedures without the droplets.

### Improved amplification uniformity of Droplet-CirSeq

**Fig. 2:**
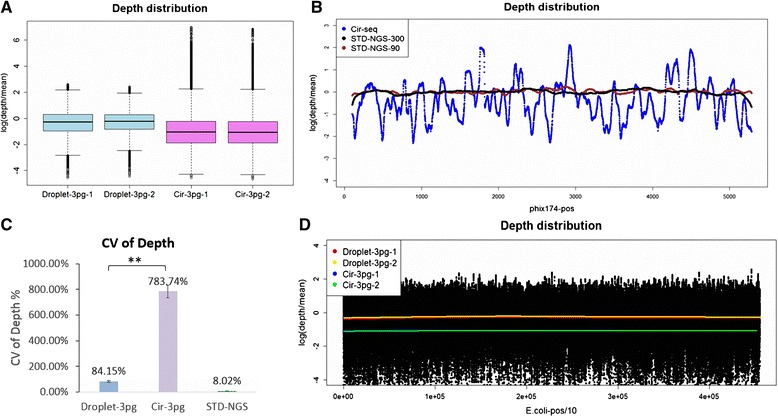
The depth distribution of Droplet-CirSeq and Cir-seq libraries. The first and last 100 bp of the genomes were excluded. **a** Boxplot of depth distribution; y axis represents the log(e) ratio of depth over mean of *E. coli* genome. Two repeats of two different types of libraries were plotted. The Droplet-CirSeq libraries (Droplet-3 pg-1 and Droplet-3 pg-2) showed more concentration depth distribution and more closeness to the mean value than the Cir-seq libraries (Cir-3 pg-1 and Cir-3 pg-2). **b** The phix174 depth distribution of the Cir-seq (90 bp fragments) and standard NGS libraries (300 bp, 90 bp fragments). **c** The depth CV (coefficient of variance) of the Droplet-CirSeq, Cir-seq, and standard NGS libraries. The Droplet-CirSeq library had a depth CV of almost 10 times less than that of the Cir-seq library (*p =* 2.6 X 10^-3^), but it was still higher than that of the standard NGS library. **d** The depth LOWESS (locally weighted scatter plot smoothing) of the entire *E. coli* genome (10 bp slide window size). The red and yellow lines represent the LOWESS of Droplet-CirSeq, while the blue and cyan lines represent the LOWESS of Cir-seq

As we known, Cir-seq is developed as an innovative method that can effectively decrease the NGS sequencing error, thereby promisingly expected to facilitate the identification of low frequency mutations [34, 35]. We assessed Cir-seq libraries using *phix174* DNA in several independent experiments (N = 9), however, it was illustrated that the ratios of read depth against the average across the whole genome ranged from 2.90 X 10^-4^ (±5.91 X 10^-4^) to 8.44 (±5.81), with a median of 0.67 (±0.15), and the depth coefficient of variance (CV) of 118.36 (±64.40)% (the depth distribution of one example showed in Fig. 2b). In contrast, the ratios for standard NGS (the insert sizes about 300 bp) lightly fluctuated around a mean of 1 (1.00791), with the depth CV of 9.63 %. These statistics obviously implicated that Cir-seq suffers from inevitable coverage bias that ascribes of the amplification preferences in the process of library preparation. The same phenomenon has been previously observed by the other researches [35, 37]. Such an inherent blemish referred as extensively amplification bias hinders its feasibility in the accurate exploitation of mutations in terms of SNPs, INDELs and copy number variations (CNVs), since uniformity and efficiency of sequencing are the most pivotal concerns when amplifying DNA molecules in vitro.

As a rule of thumb, two specific factors, in terms of the size of DNA fragmentation and the RCA reaction, may introduce amplification bias in Cir-seq. Previous studies [35, 37] have proposed that larger size of fragment specified as 90 bp had certainly improved the uniformity of sequencing coverage than the smaller one (30 bp) in Cir-seq. However, when we compared the Cir-seq libraries with the standard NGS library (both with around 90 bp insert sizes), we found the Cir-seq still suffered from severe coverage bias (*p* < 2.2 X 10^-16^) (Fig. 2b). Herein, our findings highlight that the small fragment size dedicated to the coverage bias in Cir-seq, but which is definitely not the determinant factor. Additionally, any method including Cir-seq based on RCA or MDA reaction has been reported suffering from amplification bias when the input of DNA is low, which is still the major technical challenge [38]. Based on our results and experiences, a hypothesis has been naturally proposed that the RCA system is probably the pivotal factor on account for coverage bias.

The promising strategies to overcome the barrier of coverage bias have focused on aspects in the forms of decreasing the reaction volume [39] and reducing the amplification time (or amplification fold) [40]. In our case, we made great efforts in separating the circularized DNA molecules into different compartments to reduce the uneven amplification in RCA. We aimed to eliminate intermolecular competition during amplification and uniformly confine the amplification fold by embedding the circularized DNA molecules into approximately 10 million pico-liter droplets that were similar in size. Since the available number of total droplets is limited, we tested the amplification uniformity of Droplet-CirSeq and Cir-seq libraries with 3 pg circularized *E. coli* DNA. The sequencing results showed that the depth distribution of the Droplet-CirSeq was more concentrated to the mean depth (*p* = 1.4 X 10^-3^) and the number of outliers dwindled (Fig. 2a). The CV of the Droplet-CirSeq depth was approximately 1/10 of that of Cir-seq (*p* = 2.6 X 10^-3^) but was still higher than that of standard NGS library (Fig. 2c). The LOWESS (locally weighted scatter plot smoothing) of the log(e) ratio of depth over average across the genome also showed that the depth of Droplet-CirSeq was more uniform than that of Cir-seq (Fig. 2d, Additional file 1: Figure S1), indicating that the Droplet-CirSeq significantly reduced the RCA bias by increasing the amplification probability of regions that were difficult to replicate in a tube and decreasing the amplification probability of regions that were more prone to be replicated. These results clearly demonstrated that Droplet-CirSeq significantly improved the uniformity of RCA. It is more efficient to amplify low input circularized DNA, as it allows more mutations to be identified with more accurate detected frequencies.

### Base error rate and error pattern of Droplet-CirSeq

To quantitatively assess the performances of Droplet-CirSeq and Cir-seq in the forms of error rate and error pattern, we first sequenced two disparate *E. coli* strains, namely *DH5α* and *W3110,* using standard NGS libraries on the platform of Illumina Hiseq 2500, which achieves substantial reads with coverage as high as 500 X for each strain. The extremely deep sequencing facilitates the precise identification of the variations, which could be highlighted as calibration. After exquisite data processing, we unambiguously screened out 351 polymorphic sites (6 INDELs and 345 SNPs) in the two strains.

**Fig. 3:**
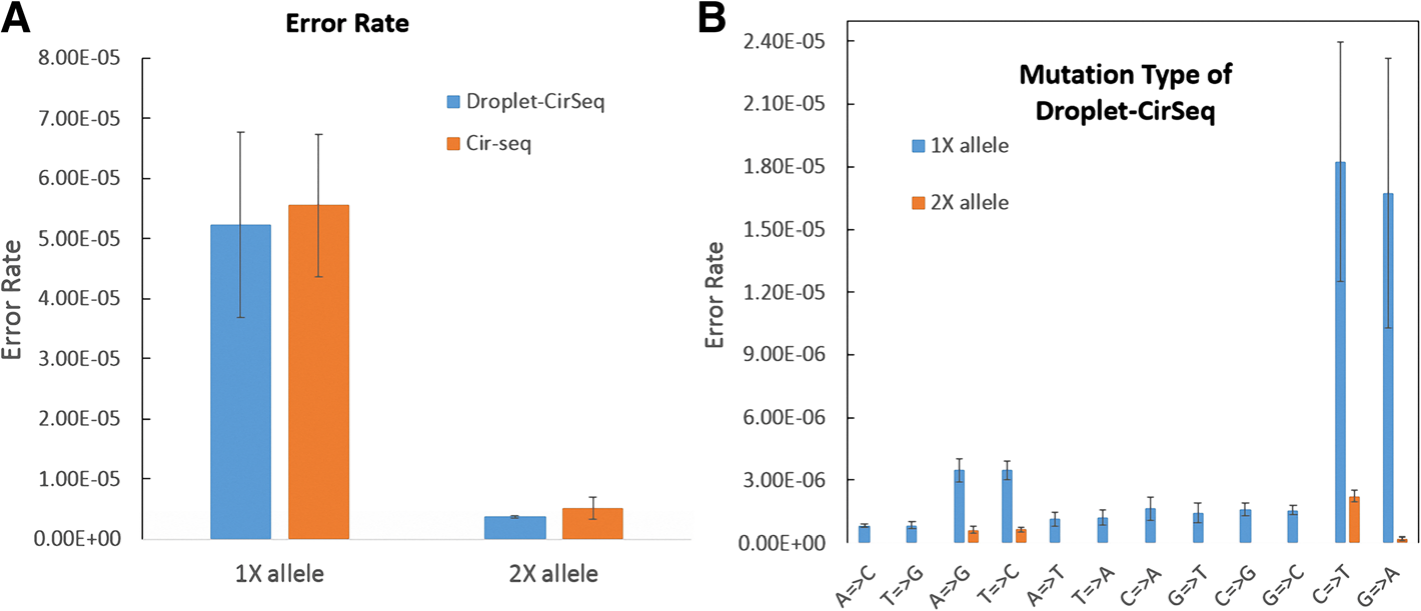
Error rate and mutation types of Droplet-CirSeq. **a** Error rate of Droplet-CirSeq and Cir-seq. The “1X allele” represents the bases that were supported at least by one circularized DNA, while the “2X allele” represents the bases that were supported by at least two different circularized DNAs. The error rate of Droplet-CirSeq was 5.23 X 10^-5^ (±1.54 X 10^-5^) at the “1X allele” criterion and 3.71 X 10^-6^ (±2.34 X 10^-7^) at the “2X allele” criterion. **b** Mutation types of Droplet-CirSeq. The error rates of most of the mutation types were lower than 3.00 X 10^-6^, but the error rates for the transitions C= > T and G= > A were almost one order of magnitude higher than the other types, and the other two transitions, A= > G and T= > C, also showed high error rates. The mutation pattern of the “2X allele” showed the same pattern as with the “1X allele”

Subsequently, we mixed the DNA of *DH5α* and *W3110* at a quantitatively specific ratio of 1:10 to simulate the circumstances of diploid with mutation frequencies of 0.1. The mixture was further sequenced using Droplet-CirSeq and Cir-seq libraries, respectively, on the platform of Illumina Hiseq 2500. As a consequence, for each library, approximately 7.5 million of 2X150 bp PE reads were obtained, aligned and utilized to determine the CSs and thereby identify the variations. Conveniently, we defined the variation that was supported by at least one CS as the “1X allele”, and the one supported by at least two different CSs as the “2X allele” aiming to further eliminate errors appearing during library preparation and sequencing. The error rates of Droplet-CirSeq and Cir-seq were calculated as the fraction of identified mutations beyond the 351 polymorphic sites, which were interpreted as the genuine variations rather than errors. Consequently, Droplet-CirSeq had an error rate of 5.23 X 10^-5^ (±1.54 X 10^-5^) when counting the “1X allele” (Fig. 3a), which was statistically comparable to that of Cir-seq (5.55 X 10^-5^ ± 1.19 X 10^-5^) (*p* = 0.69). In contrast, the error rates of Droplet-CirSeq and Cir-seq were decreased to 3.71 X 10^-6^ (±2.34 X 10^-7^) and 5.13 X 10^-6^ (±1.82 X 10^-6^), respectively, when counting the “2X allele”, where the error rate of Droplet-CirSeq presented significant lower than that of Cir-seq (*p* = 0.048).

Furthermore, we profiled the error pattern for Droplet-CirSeq. The error spectrum when counting the “1X allele” represented a dominate pattern of C- > T and G- > A, which was consistent with previous reports [34]. In addition, the error patterns of A- > G and T- > C were also higher than other types in our data. The error spectrum of the “2X allele” also showed the same pattern. Interestingly, these results provide evidence that transitions are more likely to cause errors than transversions. Spontaneous cytosine deamination may contribute to the high error rate of C- > T and G- > A, as cytosine becomes uracil through deamination [41] and then pairs with adenine, resulting in a C- > T mutation. Similarly, spontaneous cytosine deamination will cause a G- > A mutation if it occurs on the complementary strand.

### Evaluation of the efficiency of SNP calling by Droplet-CirSeq and Cir-seq

**Fig. 4:**
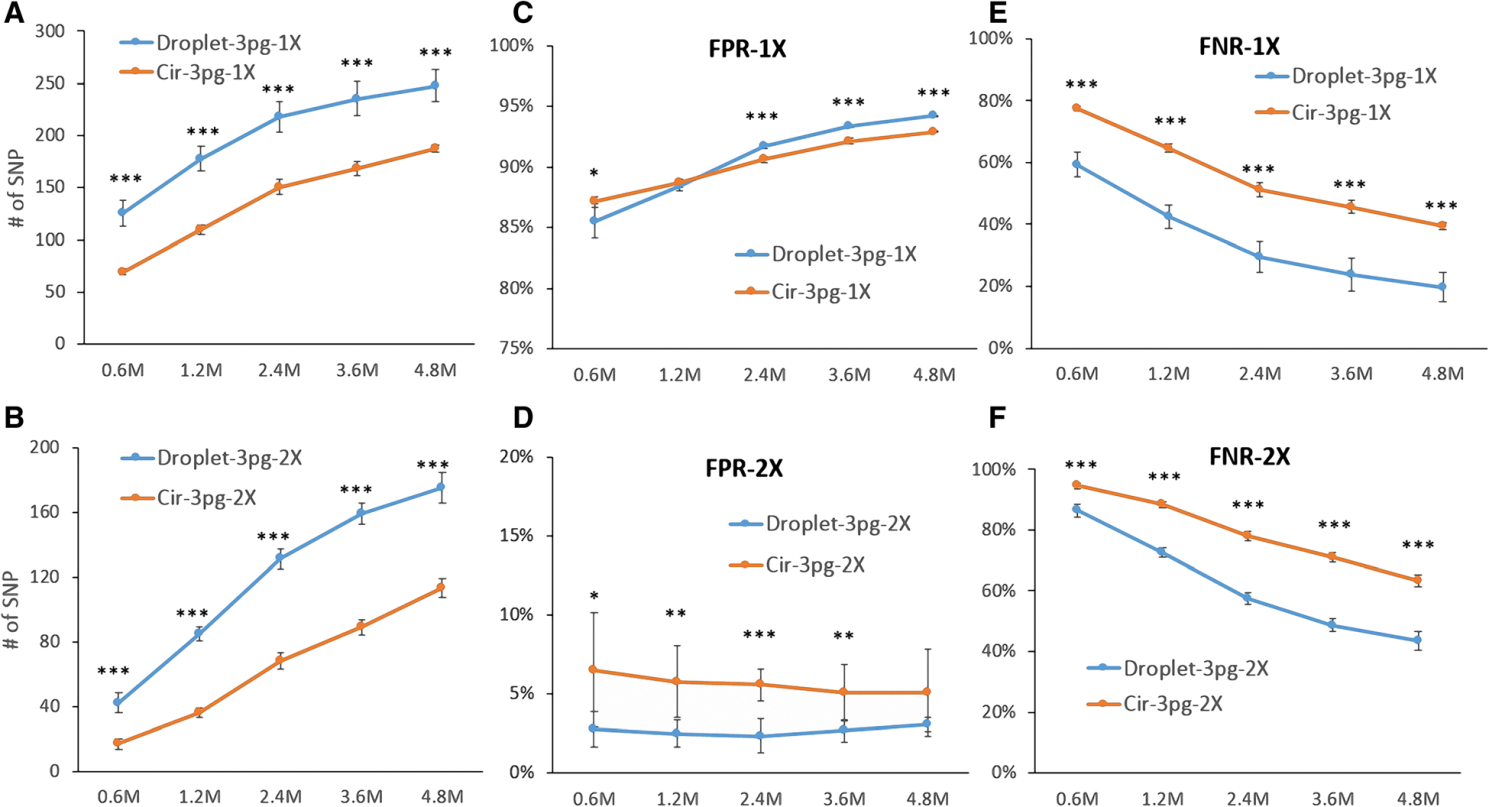
Detected mutation number, FPR and FNR of 3 pg Droplet-CirSeq and 3 pg Cir-seq libraries. **a**, **b** The number of gold standard SNPs detected by Droplet-CirSeq and Cir-seq methods with different CSs (consensus sequences) numbers at “1X allele” and “2X allele” criterion (*: 0.01 = <*p* < 0.05, **: 0.001 = <*p* < 0.01, ***: *p* < 0.001). **c**, **d** FPR of 3 pg input Droplet-CirSeq and Cir-seq libraries at “1X allele” and “2X allele” criterion. **e**, **f** FNR of 3 pg input Droplet-CirSeq and Cir-seq libraries at “1X allele” and “2X allele” criterion

Next, we evaluated the capacities of mutation detecting for Droplet-CirSeq and Cir-seq, respectively, under disparate scenarios in the mixture of two *E. coli* strains’ DNA at a quantitatively specific ratio of 1:10 (*DH5α*:*W3110*). Mostly worthy of noticing, we just took the mixture ratio of 1:10 as a specific instance to profile the influence of amplification uniformity on SNPs detection, which doesn’t constrain Droplet-CirSeq to identify the mutations with frequency of 10 %. The considered scenarios consist of varieties in read depth (CS number) simulated by proportionally sub-sampling from the total CSs (0.6 M, 1.2 M, 2.4 M, 3.6 M, 4.8 M) and in quantity of input DNA (3 pg, 30 pg, and 300 pg). As an important prerequisites, we exquisitely claimed a set of 309 highly confident sites out of 351 polymorphic sites after removing the low quality SNPs, INDELs and the common SNPs, which clearly distinguished the SNPs between the two strains and were further considered as the golden standard in the following analysis (Additional file 2: Table S1). As expected, with the increases of read depth, the identified number of SNP consistently elevated (Fig. 4a and b). Compared to the golden standard, Droplet-CirSeq averagely identified SNPs 1.3 ~ 1.8 times as more as Cir-seq when the initial DNA input was 3 pg (*p* < 0.001) (Fig. 4a) at the “1X allele” criterion. Similarly, at the “2X allele” criterion, Droplet-CirSeq also detected 1.5 ~ 2.5-fold more SNPs than Cir-seq (*p* < 0.001) (Fig. 4b).

Subsequently, we assessed the performances on the capability of SNP detection for Droplet-CirSeq with different input DNA amounts (3 pg, 30 pg, and 300 pg). The detected SNP number of 3 pg Droplet-CirSeq was comparative to those of 30 pg and 300 pg at the “1X allele” criterion (Additional file 1: Figure S2A) but less than those at the “2X allele” criterion. Intriguingly, the library with 30 pg input rather than 300 pg identified the most SNPs at the “2X allele” criterion (Additional file 1: Figure S2B), which naturally proposed a question that why 300 pg Droplet-CirSeq has not presented absolute superiority over 30 pg? According to the approximate weight of single-stranded DNA molecule, each droplet accommodates an average of 3 molecules when the DNA input is 3 pg input but up to 300 molecules when the DNA input is 300 pg. Relatively more DNA molecules accommodated in one droplet makes the spatial separation of the droplets meaningless and results in the loss of superiority. In summary, three pivotal factors, namely read depth (CS number), amount of the input DNA and the intermolecular interference, significantly influenced the SNP detection for both Droplet-CirSeq and Cir-seq, whilst, Droplet-CirSeq exhibits advanced performances on SNP identification than Cir-seq. We also noticed that 14.9 % (30 pg input) and ~19.7 % (3 pg input) positive SNPs failed to be exploited by the Droplet-CirSeq, even with a substantial CS number of 4.8 M (approximately 80X), whilst, 17.4 % (300 pg input) and ~39.2 % (3 pg input) failed to be detected by the Cir-seq with the same CS number.

The FPR (false positive rate) and FNR (false negative rate) are also essential for evaluating SNP detection methods. Herein, we calculated the FPR and FNR for Droplet-CirSeq and Cir-seq, respectively, based on the golden standard SNPs. Of note, the FPRs for both methods unfortunately reached as high as 81 % ~ 94 % with initial DNA input of 3 pg and criterion of “1X allele”, which increased with CS number, but of which the growth rate relatively slowed down when the CSs achieving 2.4 M (Fig. 4c, Additional file 1: Figure S3A). 3 pg Droplet-CirSeq presented slightly higher FPR compared to 3 pg Cir-seq (Fig. 4c), whilst, preferably owned diminished FNRs across distinct read depths. 3 pg Droplet-CirSeq showed more comparative FNRs results with the 30 pg and 300 pg (Additional file 1: Figure S3B) than 3 pg Cir-seq (*p* < 0.001). As expected, the FNR for both methods sharply dwindled with the increase of CS number, with a range of 19.8 % ~ 77.5 % (Fig. 4e), and the magnitude of reduction slowed down once the CSs exceeded 2.4 M.

However, all the aforementioned findings recapitulated that the “1X allele” criterion was inaccurate and insufficient for distinguishing the true SNPs from errors. Thereby, the “2X allele” criterion was introduced and assessed. Consequently, the FPRs for both methods drastically cut down, out of which, FPRs for 3 pg Cir-seq decreased to 5.17 % ~ 6.48 % across distinct CSs numbers, while, those for 3 pg Droplet-CirSeq dramatically diminished to 2.36 % ~ 3.04 %, significantly lower than that of Cir-seq (*p* < 0.05) (Fig. 4d). The 3 pg Droplet-CirSeq had the lowest FPR (average 2.7 %) compared to the 30 pg (*p* < 0.001) and 300 pg Droplet-CirSeq (*p* < 0.001) (Additional file 1: Figure S3C). To our surprise, the FPRs for both 3 pg Droplet-CirSeq and Cir-seq remained relatively unchanged as the increase of CS number, nevertheless, kept the augmentation for both 30 pg and 300 pg Droplet-CirSeq (Additional file 1: Figure S3C). Conversely, the FNRs certainly increased when the “2X allele” criterion was applied, out of which, FNRs for 3 pg Cir-seq ranged from 94.6 % to 63.4 %, whilst, those for Droplet-CirSeq exhibited 86.4 % ~ 43.4 %, which was very significant lower than Cir-seq (*p* < 0.001) (Fig. 4f). The 30 pg Droplet-CirSeq had the lowest FNR (29.1 % at 4.8 M CSs) compared to the 3 pg and 300 pg Droplet-CirSeq (Additional file 1: Figure S3D), and the FNRs in spite of the initial DNA input shrank with the augmentation of CSs number (Fig. 4f, Additional file 1: Figure S3D).

**Fig. 5:**
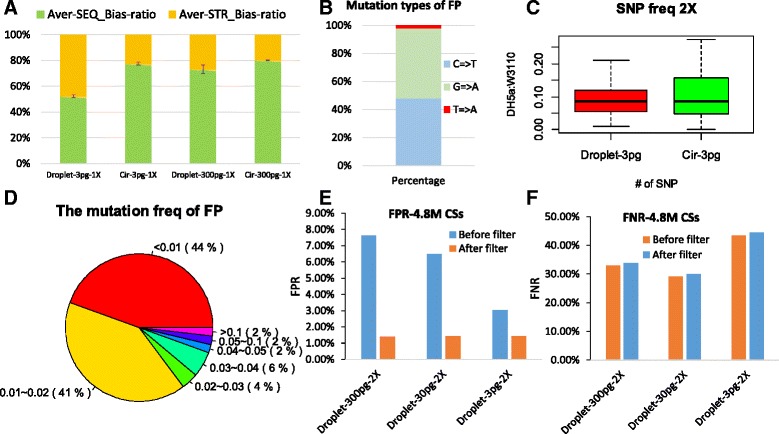
**a** FN site distribution of the Droplet-CirSeq and Cir-seq. The SEQ_Bias sites are FN sites with a depth less than or equal to 30X. The STR_Bias sites are FN sites with a depth greater than 30X but were still not detected as SNPs due to DNA strand amplification bias. The 3 pg input Droplet-CirSeq method had a lower SEQ_Bias, indicating that it improved the amplification of the poorly amplified region. The 300 pg input Droplet-CirSeq method had a lower STR_Bias, indicating that greater input improved STR_Bias. **b** Mutation type of FP sites. **c** True positive SNP frequency distribution for Droplet-CirSeq and Cir-seq; the box width indicates the detected SNP number, the outliers were excluded. **d** Mutation frequency of FP sites. **e** FPR of different input Droplet-CirSeq libraries after filtering with the mutation frequency pattern. **f** FNR of different input Droplet-CirSeq libraries after filtering with the mutation frequency pattern

Based on the above findings, a technical puzzle about Droplet-CirSeq has been literally proposed, why FNR still remains considerable and why Droplet-CirSeq still accommodates FP (false positive) sites after filtering with the “2X allele” criterion. To conveniently address this question, we refer to the FN (false negative) sites whose depths were less than or equal to 30X as the SEQ_Bias (sequence-bias) sites when the average depth was 80X, due to their sequence context interaction in the process of amplification in the RCA reaction, and the FN sites with depths over 30X as the STR_Bias (strand-bias) sites, due to the uneven amplification of different DNA strands. The 3 pg Droplet-CirSeq FN sites were composed by 52.3 % SEQ_Bias sites and 47.7 % STR_Bias sites, whilst, the 3 pg Cir-seq FN sites were characterized by 77.6 % SEQ_Bias sites and 22.4 % STR_Bias sites (Fig. 5a). The relatively lower SEQ_Bias of the 3 pg Droplet-CirSeq indicated its improvement of amplification bias by enhancing the amplification capacity of the poor replicate regions, however, there were still certain virgin regions remained. In addition, comparing the 3 pg and 300 pg Droplet-CirSeq libraries, the latter had less STR_Bias sites, which suggested that more input DNA could decrease STR_Bias. Such results were also concordant with the previous conclusion that more input DNA and less intermolecular interference are effective on generating more uniform amplification and detecting more true SNPs. In other words, the FNR was influenced by the uniformity of the amplification and the input DNA quantity. The Droplet-CirSeq method has advanced the uniformity of amplification, and its performance would be further ameliorated when more DNA is added to more droplets.

In reference to the FP sites, there were totally 62 FP sites in all libraries (8 Droplet-CirSeq libraries). Out of them, we have to say, there might be genuine SNPs, which are actually supported by only 1 ~ 2 reads but treated as random errors in the standard NGS data process due to the ultra-low frequency. We investigated and revealed 11 sites under such a circumstance. Additionally, there is one common site at *E.coli*: 4294083 shared by each Droplet-CirSeq, which was actually a heterozygous site but failed to be detected by bcftools. Besides this common site, the other 54 FP sites richly represented in two types of base substitutions, namely G to A (50.0 %) and C to T (48.1 %) (Fig. 5b).

Then, we profiled the mutation spectrum of TP (true positive) and FP sites for Droplet-CirSeq and Cir-seq, respectively. The frequencies of TP sites for both methods in the case of 3 pg DNA input were all averaged at the true frequency (0.1), while, Droplet-CirSeq presented more concentrated and representative of the true frequency than Cir-seq libraries (*p* = 4.8 X 10^-3^) (Fig. 5c). Majority of TP sites exhibited relatively high frequencies, and only 1.8 % had frequencies lower than 0.02. In contrast, 85.2 % of FP sites were characterized by lower frequency (<=0.02), where, 44.4 % had frequencies lower than 0.01, and 40.8 % were between 0.01 and 0.02 (Fig. 5d). The distinguishable frequency spectrums between TP and FP sites hint that FP sites could be partially filtered out according to the frequency pattern. In our case, after filtering out mutations with frequency less than 0.02, the FPR was significantly decreased to 1.42 % for the Droplet-CirSeq libraries (Fig. 5e), whilst the FNR remained nearly the same (Fig. 5f). It is also noted, compare to the average sequencing depth of the genome (80X), 90.7 % of the FP sites inhabited in the regions with depths higher than 100X, which were prone to amplification in the RCA reaction. Additionally, for the different DNA input amounts for Droplet-CirSeq, the 3 pg Droplet-CirSeq had the lowest FPR before filtering and showed the least change in FPR after filtering, indicating the amplification of the 3 pg Droplet-CirSeq was the most uniform.

## Discussion

Cir-seq reduces high throughput DNA sequencing errors by linking the tandem copies of one DNA fragment in one reaction of paired-end sequencing but suffers from severe imbalance of amplification. To avoid such blemishes, we developed an innovated method termed Droplet-CirSeq. The Droplet-CirSeq approach combines millions of picoliter droplets and the Cir-seq method for uniform and ultrasensitive detection of rare mutations. The picoliter droplets are used to eliminate the intermolecular competition of DNA molecules and thus improve the efficiency of the amplification of the poorly replicated region. On the other hand, picoliter droplets provide an equal volume of reagent and the byproduct pyrophosphate, together with the uniform size and infinitesimal volume of the droplets, to confine the final amplification yields of the regions which were more prone to replication. These advantages allow this Droplet-CirSeq method to amplify circularized single-stranded DNA with much less bias and detect rare mutations more effectively and precisely than other methods. Droplet-CirSeq has the error rate of 3 ~ 7 X 10^-5^ for SNPs with one consensus sequence (1X allele), and this was decreased to 3 ~ 5 X 10^-6^ when the SNPs were supported by two consensus sequences with different breakpoints (2X allele).

The error rate of Droplet-CirSeq at the “2X allele” criterion, which is close to the limitation of detected mutations in vitro based on NGS methods [33, 34]. Under this low frequency, the DNA damage errors produced during DNA extraction and preparation became the dominate error source, and these errors are difficult to distinguish from the genuine low frequency mutations. Fortunately, Droplet-CirSeq is quite suitable for low input DNA and, therefore, provides an alternative solution for detecting ultra-low frequency mutations by reducing population size. For example, one mutation in a large tumor nodule with a frequency of 1X10^-5^ cannot be detected by current methods. However, we can sample the tumor several times by taking a small number of cells so that the mutation frequency in some samples will increase to a detectable level. Small population size implies low DNA quantity, which has limited mostly current low-frequency detection methods (such as ref 33, 34) but is suitable for Droplet-CirSeq. The ultra-sensitive and uniform detection of mutations by Droplet-CirSeq with low input DNA makes it the competitive method for successfully detecting mutations in small populations with low quantity DNA.

To understand the relationships between and among input circularized DNA, droplet number, amplification uniformity and SNP detection capability, we tested different input DNA amounts (3 pg, 30 pg, and 300 pg) for Droplet-CirSeq library preparation using approximately 5 ~ 10 million droplets. Three picogram Droplet-CirSeq obtained the best amplification balance (*p* < 0.01), while 300 pg Droplet-CirSeq obtained the worst (*p* < 0.001) (Additional file 1: Figure S4). This suggests that increasing the amount of DNA within one droplet decreased amplification uniformity. However, the results of mutation detection demonstrated that 30 pg Droplet-CirSeq identified the most genuine mutations. In general, the capacity of mutation detection is determined by two factors, the input DNA quantity, and the uniformity of the amplification. Thirty picogram Droplet-CirSeq may have the best tradeoff between input DNA quantity and amplification uniformity. Droplet volume is also critical for proper amplification. If the droplet volume is too large (such as a nanoliter or microliter), it will not effectively inhibit the region that is prone to amplification. If the droplet volume is too small, it will lead to insufficient amplification or other unpredictable problems. Collectively, increasing the droplet number, reducing the amount of circularized DNA contained in each droplet and decreasing the droplet volume appropriately will result in better amplification.

In this study, we mixed two *E. coli* strains (*DH5α* and *W3110*) at a ratio of 1:10 to compare the uniformity of the amplification and capability of mutation detection for Droplet-CirSeq and Cir-seq. The results of this study clearly showed that Droplet-CirSeq has a great advantage over Cir-seq in detecting mutations. This advantage of Droplet-CirSeq is diminished when it is compared with the standard NGS methods because of its relatively unbalanced amplification and double correction scheme; however, the advantage of Droplet-CirSeq over standard NGS methods is incomparable when it is used to detect low frequency mutations. We also mixed two *E. coli* strains at a ratio of 1:100, the “2X allele” analysis revealed a FPR of 25 %, out of which, two dominant substitutions C = > T and G = > A accounted for 78.6 %. This phenomenon was consistent with the error pattern of Droplet-CirSeq and has also been reported by many other previous studies [8, 32–35]. Worthy of noting, Droplet-CirSeq still remains considerable poorly amplified regions (approximately 10 % in *E. coli*), which ascribes of inherent characteristics of RCA and this pattern was repeatable and stable.

Compared with Cir-seq, the Droplet-CirSeq approach has more uniform amplification, more accurate mutation frequency detection, a lower false positive rate, and a lower false negative rate. Droplet-CirSeq will be one of the best choice for detecting mutations at frequencies ranging from 10^-4^ to 10^-1^. Droplet-CirSeq has an unparalleled advantage for low input DNA. Droplet-CirSeq can be widely used for detecting low frequency mutations of circulating DNA in the plasma, DNA of uncultured microorganisms, DNA of cancer subclones and DNA of tumor-associated niche cells. It can also be used to filter out the errors in degenerated ancient DNA and formalin-fixed, paraffin-embedded DNA samples, et.al.

## Conclusions

In this study, we described a novel approach namely digital droplets-based circle sequencing, abbreviated as Droplet-CirSeq, which is able to detect low or ultra-low frequency mutations with greatly reduced amplification bias. Briefly, it introduces a pico-liter amplification system to Cir-seq to improve the amplification bias. As expected, the coefficient of variance of read depth for Droplet-CirSeq was merely 1/10 of that of Cir-seq, and the identified allele frequency presented more concentrated to the authentic frequency of mixtures, illustrating a significant improvement of amplification bias and accuracy in allele frequency determination. In addition, compared to Cir-seq, our method owns the competitive characteristics with an incredibly low error rate of 3 ~ 5 X 10^-6^, capability of rescuing 2.5 times of genuine SNPs at the same sequencing depth, a lower false negative rate, and an even lower false positive rate. Intriguingly, Droplet-CirSeq can successfully take pico-gram level of DNA as input. With the particular characteristics and competitive features, Droplet-CirSeq would be promisingly and widely used in the identification of low frequency mutations initiated from extremely low input DNA, such as circulation DNA of plasma, subpopulation within tumor et al.

### Availability of supporting data

The data sets supporting the results of this article are available in the NCBI Sequence Read Archive (SRA) repository, [http://www.ncbi.nlm.nih.gov/sra] under accession number: PRJNA285951.

## Additional files

Additional file 1: Supplemental figures S1-S4. Comparations of depth distribution, detected SNP numbers, FPR and FNR between Droplet-Cirseq and Cir-seq. (PDF 623 kb) Additional file 2: Supplemental Table S1. W3110 and DH5α specific polymorphic sites. (XLSX 55 kb)

## Abbreviations

CNVs: copy number variations
CSs: consensus sequences
CV: coefficient of variance
FNR: false negative rate.
FPR: false positive rate
INDELs: small insertions and deletions
LOWESS: locally weighted scatter plot smoothing
MDA: multiple displacement amplification
NGS: next generation sequencing
PE: pair end
RCA: rolling circle amplification
