# Erratum to: ‘Ultra-precise detection of mutations by droplet-based amplification of circularized DNA’

**DOI:** 10.1186/s12864-016-2683-5

**Published:** 2016-06-02

**Authors:** Kaile Wang, Qin Ma, Lan Jiang, Shujuan Lai, Xuemei Lu, Yali Hou, Chung-I Wu, Jue Ruan

**Affiliations:** Key Laboratory of Genomic and Precision Medicine, Beijing Institute of Genomics, Chinese Academy of Sciences, Beijing, China; University of Chinese Academy of Sciences, Beijing, China; State Key Laboratory of Biocontrol, School of Life Sciences, Sun Yat-Sen University, Guangzhou, China; Department of Ecology and Evolution, University of Chicago, Chicago, USA; Agricultural Genomics Institute at Shenzhen, Chinese Academy of Agricultural Sciences, Shenzhen, China

Unfortunately, the original version of this article [1] contained an error. The acknowledgements was included incorrectly. The correct acknowledgements can be found below:
