# Supplementary material for: Ultra-precise detection of mutations by droplet-based amplification of circularized DNA
Source: BMC Genomics. 2016 Mar 9;17:214. doi: 10.1186/s12864-016-2480-1 (PMC4784281; doi:10.1186/s12864-016-2480-1)
Supplement: Additional file 1: — Supplemental figures S1-S4. Comparations of depth distribution, detected SNP numbers, FPR and FNR between Droplet-Cirseq and Cir-seq. (PDF 623 kb) [file 12864_2016_2480_MOESM1_ESM.pdf]

SUPPLEMENTARY DATA

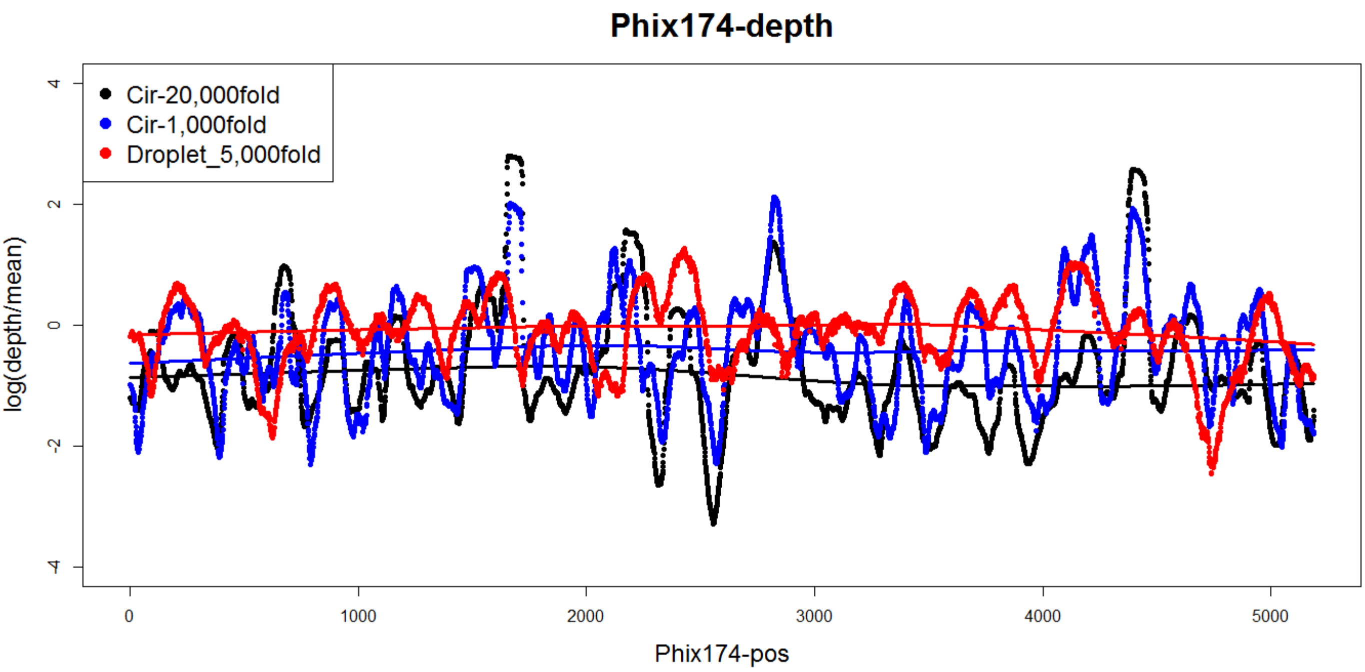

Fig. S1. Depth distribution of Droplet-CirSeq and Cir-seq libraries. The first and last 100 bp of the genomes were excluded. Wave lines represent the depth distribution, and the smooth curves represent the LOWESS (locally weighted scatter plot smoothing) of the depth. The uniformity of the Cir-seq improved when the amplification fold was reduced, but Droplet-CirSeq obtained better results, even with greater amplification fold.

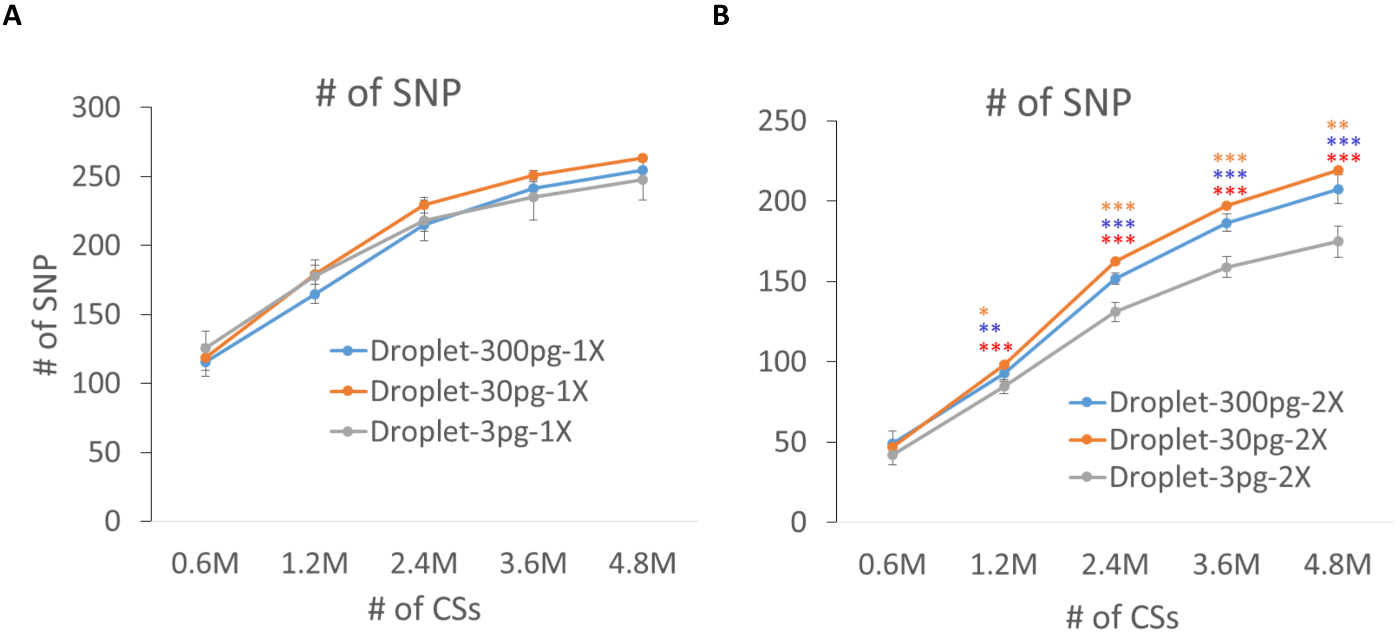

Fig. S2. Detected mutation number of different input Droplet-CirSeq libraries at 1X and "1X allele" criterion. *(A)* *p*-values between 3pg input and 30pg input at different number of CSs were all greater than 0.05, and *p*-values between 3pg and 300pg also greater than 0.05 at "1X allele". *(B)* *p*-values between 3pg and 30pg, 3pg and 300pg at 0.6M CSs were greater than 0.05, but when CSs number reached to 1.2M, *p*-values between 3pg and 30pg, 3pg and 300pg were less than 0.01, and

between 30pg and 300pg were less than 0.05 at "2X allele". These results indicated 3pg Droplet-CirSeq detected SNP number was comparative to 30pg and 300pg Droplet-CirSeq at "1X allele", but less than those at 2X criterion, and 30pg detected most SNPs. The red asterisks were the *p*-value between 3pg and 30pg, the blue asterisks were the *p*-value between 3pg and 300pg, and the brown asterisks were the *p*-value between 30pg and 30pg.

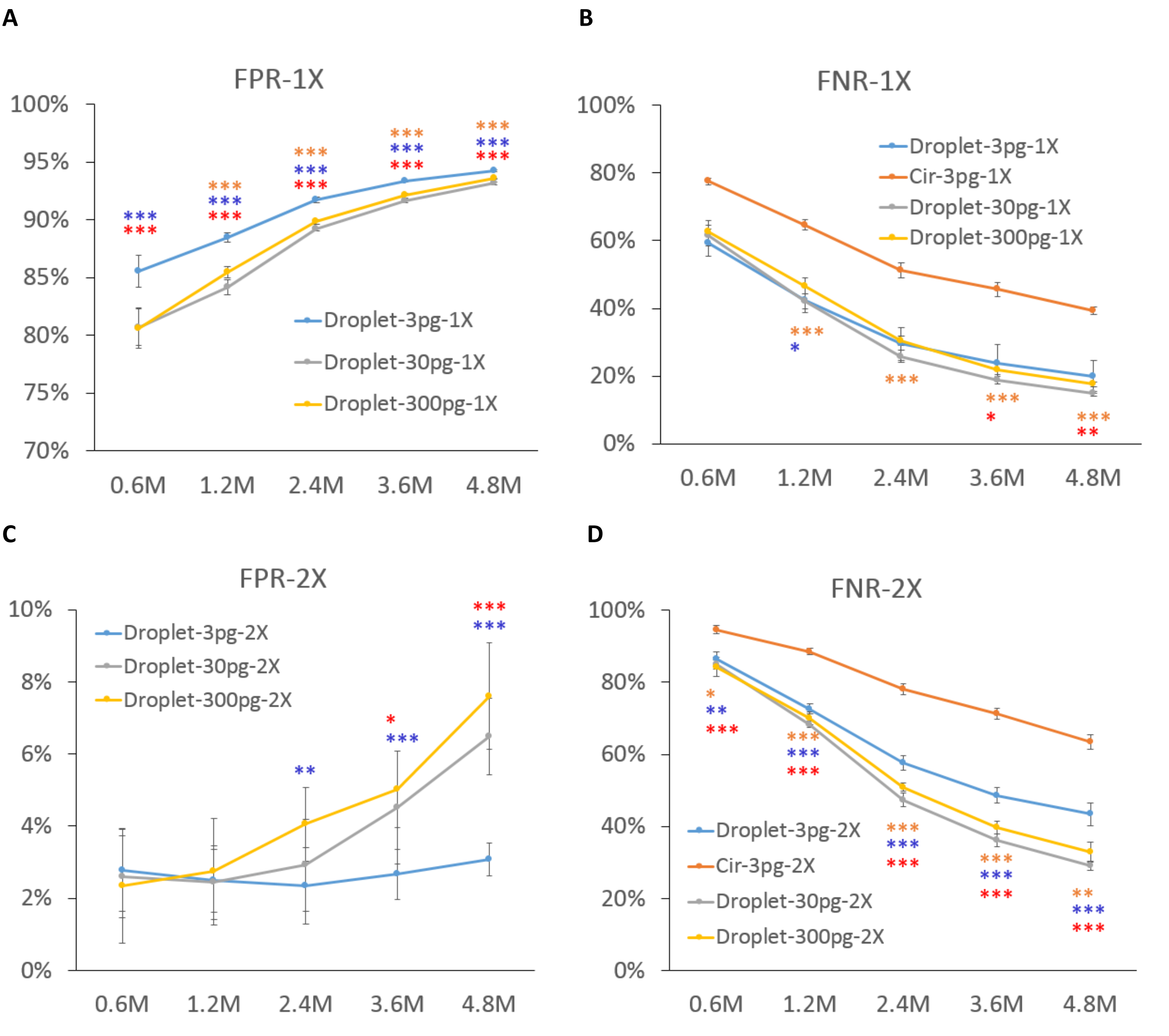

Fig. S3. FPR, FNR of Droplet-CirSeq and Cir-seq libraries. *(A, B, C, D)* FPR and FNR of different input Droplet-CirSeq and Cir-seq libraries at 1 X and "2X allele" criterion. The red asterisks were the *p*-value between 3pg and 30pg, the blue asterisks were the *p*-value between 3pg and 300pg, and the brown asterisks were the *p*-value between 30pg and 30pg.

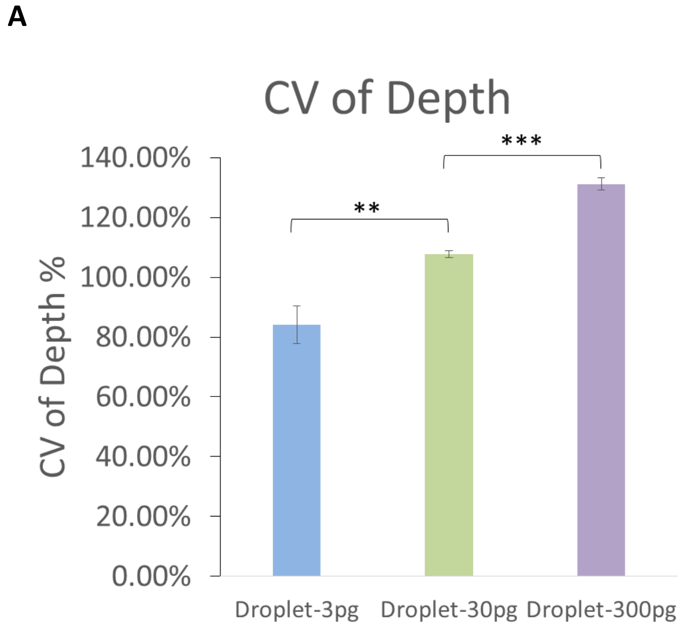

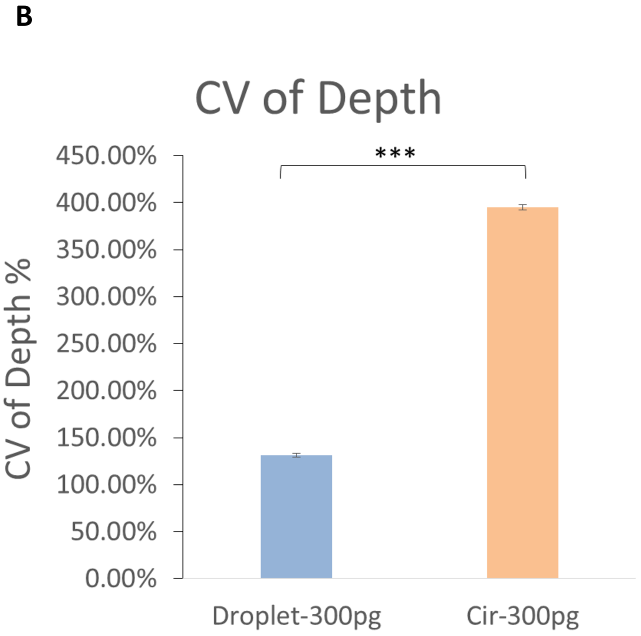

Fig. S4. Depth distribution of Droplet-CirSeq and Cir-seq libraries. The first and last 100 bp of the genomes were excluded. *(A, B)* The depth CV (coefficient of variance) of the different input (3 pg, 30 pg, and 300 pg) Droplet-CirSeq libraries and the 300 pg Droplet-CirSeq and 300 pg Cir-seq libraries.
